# A New Compressed Data Acquisition Method for Power System Based on Chaotic Compressive Measurement

**DOI:** 10.3390/s24237499

**Published:** 2024-11-25

**Authors:** Shan Yang, Zhirong Gao, Jingbo Guo

**Affiliations:** 1 College of Electrical and Information Engineering, Hunan University of Technology, Zhuzhou 412007, China; gao_zhirong@126.com; 2 Department of Electrical Engineering, Tsinghua University, Beijing 100084, China; guojb@tsinghua.edu.cn

**Keywords:** data acquisition method, new power system, compressed sensing, chaotic measurement matrix, compressed acquisition

## Abstract

The digitalization level of the new power system driven by “dual carbon” is increasing, leading to a growth in the amount of data that need to be acquired. This has intensified the contradiction between data volume and acquisition capacity. Therefore, it is urgent to study compressed data acquisition methods for power systems based on data compression. In this regard, a novel compressed data acquisition method based on chaotic compressive measurement with the compressed sensing principle is proposed. Firstly, the advantages of applying compressed sensing are analyzed for data acquisition in power systems, and the key issues that need to be addressed are identified. Subsequently, a chaotic map is sampled based on the basic requirements of the measurement matrix in compressed sensing, and the chaotic compressive measurement matrix is constructed and optimized based on the sampling results. Next, the sparse data difference of the power system is used as the compression target for the optimized chaotic measurement matrix, and an acquisition process is designed to recover the complete power data from a small amount of compressed data. Finally, the proposed method is validated in a case study, and the results demonstrate that the method is correct and effective.

## 1. Introduction

In order to achieve the major goals of “carbon peaking” and “carbon neutrality”, the power system in China has integrated “low-carbon” operational requirements into the construction of a new power system. This marks China as entering a new period of “low-carbon” electricity [1]. Under the drive of the “dual carbon” strategy, the new power system has identified “highly digitized“ as its main construction direction. This has initiated the comprehensive digital transformation of the power system [2]. Therefore, the key technologies of the “highly digitized” new power system have significant research value.

In the process of constructing a “highly digitalized” new power system, the breadth, frequency, depth, and dimension of power system will continue to increase in order to establish the complete process of “dual carbon” operation in the power system. This inevitably leads to further expansion of the data scale. The contradiction between the data acquisition capability of the power system and “low-carbon”, as well as the “digitization” of the system, continues to escalate, and further intensifies the challenges of data acquisition brought by large-scale data [3,4]. Therefore, it is necessary to further study the data acquisition methods of the power system.

There are two main approaches to the research on data acquisition methods in the power system at present. The first approach focuses on enhancing the communication capabilities of large-scale data. This involves improving existing communication methods or introducing emerging ones to enhance the transmission efficiency of the data acquisition method and to ensure the rapid acquisition of big data. Reference [5] utilizes LoRa and 4G communication technologies to transmit various types of power data collected by the power monitoring system to the internal network, realizing real-time collection and transmission of power data. This solution has advantages such as high data accuracy, long transmission distance, and easy scalability. Reference [6] takes power line communication as the communication mode, which is widely used in power systems. Considering the impact of communication capacity on real-time data acquisition, the positioning of data collectors is re-planned to ensure real-time access to power data while minimizing installation costs. Reference [7] integrates Industrial Internet of Things (IIoT) with smart grid data acquisition, and utilizes the powerful real-time performance and high reliability of IIoT to enhance the data acquisition capability of the power system. Reference [8] integrates cloud computing with data acquisition in a power system, and utilizes cloud servers to collect and process massive amounts of data from various power terminals. Subsequently, the data reception end of the power grid acquires the data through the cloud.

The data acquisition method, starting from improving communication capabilities, is merely an auxiliary method or remedial measure to reduce the impact of big data on real-time acquisition of power system data. Therefore, the second approach in the data acquisition method focuses on the data themselves, based on data dimensionality reduction processing, which alleviates the pressure of data acquisition. The basic idea of this approach is based on the spatial transformation of data, transforming a large amount of data into a small amount of key information, thereby achieving the compressed acquisition of big data. Reference [9] proposes a smart grid data compression method using wavelet transform. This method calculates the optimal basis for the data using weighted entropy and reconstructs the data based on thresholded detail coefficients and unthresholded approximation coefficients. Reference [10] proposes a data compression method for power quality based on independent component analysis and Fast Fourier Transform (FFT). This method represents the signal using several sets of different independent components and transforms the independent components using the FFT algorithm. Reference [11] proposes a high-performance algorithm for power quality data compression based on wavelet transform. This method applies a six-level transform of a biorthogonal wavelet and utilizes a normalization technique to correct the error between the original data and the reconstructed data. Reference [12] proposes a smart meter data compression method based on deep learning. This method utilizes the unique characteristics of smart meter readings to design an efficient and lightweight autoencoder for extracting feature efficiently. Reference [13] proposes an online compression method for household power data based on time series. This method adaptively segments the data and extracts key information as compressed data using a similarity measure based on segment statistical distance. Reference [14] proposes a data compression method for smart power distribution systems based on singular value decomposition. This method is simple and user-friendly, and can achieve a good balance between data compression and information loss, thereby significantly reducing the amount of data transmitted through communication networks. In [15], a data compression method based on Tucker tensor decomposition is proposed. The proposed method takes advantage of organizing time series into multidimensional arrays (tensors) to achieve higher compression ratios while preserving most of the original data attributes in order to lower reconstruction error. In [16], a data loss compression method based on optimal singular value decomposition (OSVD) is proposed. By solving for the optimal singular values of the power data based on the quality of the data and compression ratio requirements, the method achieves data compression by appropriately neglecting other singular values.

The above data acquisition methods for power systems start with direct data compression, and can be accomplished based on existing communication infrastructure, which better adapts to the ongoing issue of data growth. However, compression-based data acquisition methods often face a trade-off between high compression and high accuracy. On one hand, the fewer the data that are collected after compression, the more information is sacrificed, resulting in lower data acquisition accuracy. On the other hand, integrating other data processing methods can improve data acquisition accuracy, but it also increases the complexity of data acquisition.

The compressed sensing theory proposed by Donoho et al. provides a new approach for compressed data acquisition in power systems. Compressed sensing theory consists of three components: sparse representation [17,18,19], compressive measurement, and recovery reconstruction. Its essence is to directly measure the “information” of data by the compressive measurement matrix. Due to the information content being much smaller than the data volume, data compression can be achieved. Therefore, the data compression in power systems requires that the compressed measurement matrix aligns with practical applications.

The data compression of power systems is completed at the local end, while the data recovery is performed at the control center. It is required that the compressive measurement matrix has deterministic characteristics. However, traditional measurement matrices are primarily random measurement matrices [20,21,22], which do not meet the application requirements of power systems. Therefore, this paper uses chaotic mapping with pseudo-randomness [23,24,25] to construct a measurement matrix suitable for power systems. The advantage of the chaotic compressive measurement matrix lies in its ability to simultaneously meet the requirements of randomness and determinism for the measurement matrix. If the elements of the measurement matrix are derived from a specific chaotic mapping equation, then the measurement matrix is uniquely determined.

This paper proposes a compressed data acquisition method based on chaotic compressive measurement. Compared to spatial-transformation-based compressed acquisition, this approach has two advantages. First, the compression approach is more advanced. It combines the principle of compressed sensing with data acquisition, directly utilizing the compressive measurement matrix to achieve data compression without losing information during the compression process. Second, the acquisition accuracy is higher. The data acquisition process only compresses the amount of data, not the information. The data restored from complete information results in smaller collection errors.

The remaining sections of this article are as follows. Section 2 introduces the necessity of applying compressive sensing theory to the compressed data acquisition of new power systems, as well as the practical problems that need to be addressed in data acquisition based on compressive sensing. Section 3 introduces the construction and optimization methods of the chaotic compressive measurement matrix, as well as how to use the optimized measurement matrix to achieve compressive measurements of data differences and compressed data acquisition in power systems. Section 4 uses a power grid case to calculate the compression performance and acquisition accuracy of the proposed compressed data acquisition method and to analyze the influencing factors in compression performance and acquisition accuracy. Section 5 is the conclusion of this paper.

## 2. Compressed Data Acquisition Based on Compressed Sensing

### 2.1. The Necessity of Implementing Data Acquisition Based on Compressed Sensing Principle

**Figure 1:**
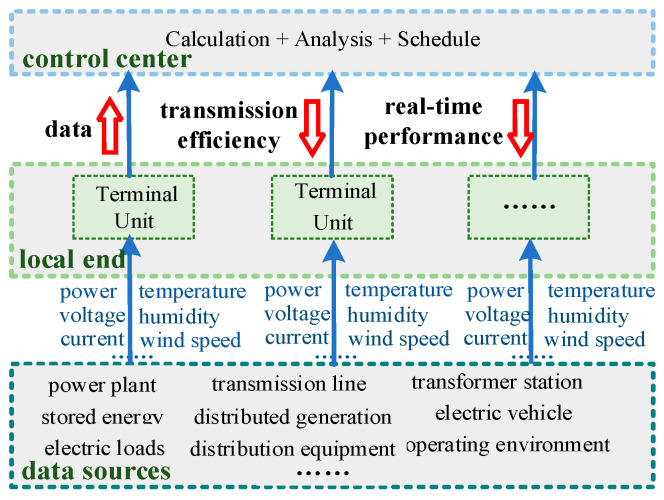
Power system data acquisition process.

The data acquisition in the power system refers to the process of collecting various types of data generated at the local end during operation and transmitting it to the control center through the communication system. For the power system, there are two main sources of big local data. One source comprises electrical variables (such as voltage, current, power, etc.), which have been widely present in traditional power systems. These data form the most fundamental and crucial data resources in the new power system. The other source comprises the important monitoring variables (such as temperature, humidity, wind speed, etc.) involved in maintaining the normal operation of the power system. Under the development trend of the new power system, the sharp increase in these two types of data will have a significant impact on the power system. Figure 1 illustrates the data acquisition process in the power system.

The volume of data in the new power system is enormous and shows an upward trend. The increasing number of nodes, the large-scale integration of renewable energy, and the timely response of user demands require a large amount of data for in-depth analysis of the power operation status. With the gradual proliferation of smart meters and sensors, more data will be generated at the local end. The sharp increase in data will have a serious impact on the power system.

It can be seen from Figure 1 that the increase in local data has two main impacts on the power system. Firstly, when the data increase but the communication capacity is not promptly upgraded, this leads to a decrease in data transmission efficiency. Secondly, the increase in data weakens the timeliness with which data are received by the control center, resulting in a decrease in decision-making efficiency at the control center. To mitigate the impact of increased local data on the normal operation of new power systems, this paper studies a data acquisition method for power systems based on data compression.

**Figure 2:**
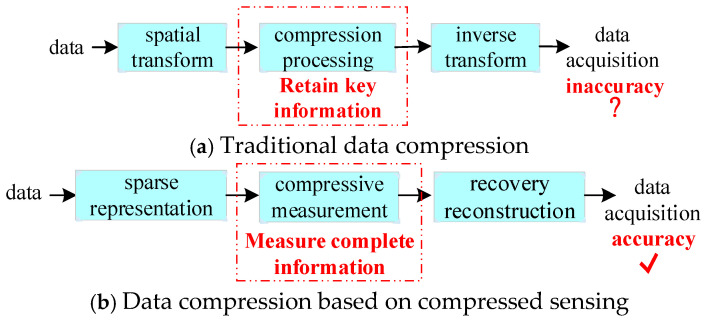
Comparison between traditional data compression and compressive-sensing-based data compression.

It is worth noting that, unlike traditional compressed data acquisition methods based on spatial transformation, this paper realizes compressed data acquisition for power systems based on compressive sensing. The principles of these two methods are compared in Figure 2.

Compared to traditional data compression, compression based on compressive sensing has more advantages. Traditional compression is a lossy processing method that only retains the main information. On the other hand, compressive-sensing-based compression directly senses all the information in the data, with the amount of information based on the data being much smaller than the data themselves, achieving significant data compression. This type of data compression can preserve complete data information while maintaining high compression and high accuracy. Therefore, it is necessary to construct a compressed data acquisition method for power systems based on the theoretical foundation of compressive sensing.

### 2.2. Research Ideas on Compressed Data Acquisition Method Based on Compressed Sensing

#### 2.2.1. Mathematical Expression of Compressed Sensing Principle

For any compressible discrete datum s with length *N*, its compressed sensing process consists of three parts: sparse representation, compressive measurement, and recovery reconstruction [17,18,19].

In sparse representation, the data ***s*** can be represented by a sparse basis ***Ψ*** and sparse coefficients ***θ***. The number of non-zero elements in ***θ*** is *K*, and *K* << *N*.

For compressive measurement, if the data ***s*** can be sparsely represented, then it is possible to perform the compressive measurement on data ***s*** using a measurement matrix ***Φ*** ∈ ***R****^M^^×^^N^*, resulting in a measurement vector ***y*** with length *M*. Since *M* < *N*, the compressed data length will be much smaller than the original data length.

Recovery reconstruction is a method to recover the original data ***s*** from the compressed data ***y***. Recovery reconstruction based on compressed sensing can be expressed by solving the optimization problem under the *l*_1_ norm.

Therefore, the specific expression of compressed sensing is as in (1):

(1) $$\left\{\begin{array}{l} s=\Psi \theta \\ y=\Phi s\\ \hat{\theta }=argmin{\left\|\theta \right\|}_{1}\begin{array}{cc} & \begin{array}{cc} s.t. & y=\Theta \theta \end{array} \end{array} \end{array}\right.$$

where ***Ψ*** = [***Ψ***_1_, ***Ψ***_2_, *…*, ***Ψ**_N_*], ***θ*** = [***θ***_1_, ***θ***_2_, *…*, ***θ**_N_*]^T^, $\hat{\theta }$ represents the optimal computed value of the sparse coefficient ***θ***, and ***Θ*** = ***ΦΨ*** represents the sensing matrix, which satisfies the Restricted Isometry Property (RIP).

From the compressed sensing expression in (1), it can be seen that the key to combining compressed sensing with the data acquisition of new power systems is to construct ***Ψ*** and ***Φ*** that are suitable for the data characteristics of the power system, and to ensure that ***Θ*** satisfies RIP.

#### 2.2.2. Key Issues in Compressed Data Acquisition Based on Chaotic Compressive Measurement

**Figure 3:**
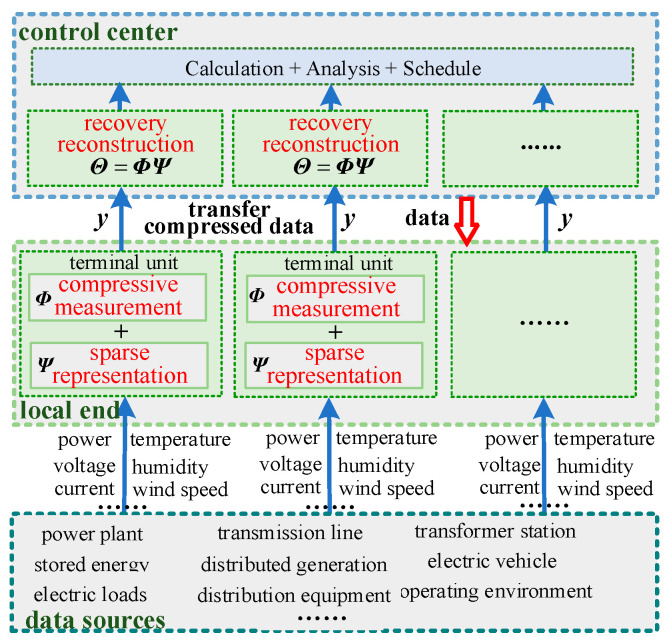
Compressed data acquisition process of power system based on the compressive sensing principle.

Based on the principle of compressive sensing and the data acquisition process of the power system shown in Figure 1, a combined approach is designed. Firstly, the power data are compressed based on sparse representation and compressive measurement at local end. Next, the compressed data y are transmitted to the control center through communication system. Finally, the power system data before compression are restored based on recovery reconstruction in the control center. The specific compressed data acquisition process of power system based on the compressive sensing principle is shown in Figure 3. The text highlighted in red in Figure 3 indicates the key steps of the data acquisition process proposed in this paper, the blue upward arrows represent the direction of data flow, and the red downward arrow indicates a reduction in data volume.

It can be seen from Figure 3 that the main feature of compressed data acquisition based on compressive sensing principle is that the data compression is performed at the local end and the data recovery is completed at the control center, both requiring same sparse basis ***Ψ*** and measurement matrix ***Φ***. To maintain consistency between ***Ψ*** and ***Φ*** at both ends, the transmission of ***Ψ*** and ***Φ*** becomes a concern. Therefore, compressed data acquisition based on compressed sensing needs to address how to avoid the transmission of ***Ψ*** and ***Φ*** while still allowing the control center to obtain complete acquisition data.

In order to solve the problem of transmitting ***Ψ*** and ***Φ*** caused by data compression and recovery being set at different ends in the power system, this paper designs the deterministic ***Ψ*** and ***Φ*** as known parameters for the compressed acquisition method. By providing the same ***Ψ*** and ***Φ*** at the local end and the control center, the transmission process is avoided. The design idea for ***Ψ*** and ***Φ*** is as follows:

Firstly, the sparse basis ***Ψ*** directly adopts the unit matrix. Due to the short data acquisition interval in power systems, the data do not change significantly within a short period of time, resulting in a large number of zero elements in the difference vector of the local data at adjacent time. Therefore, the data difference vector at adjacent time is a kind of compressible data, which can be utilized for compressive measurement. In this case, ***Ψ*** is the unit matrix.

Secondly, the compressive measurement matrix ***Φ*** is constructed by chaotic mapping. By combining the advantages of both deterministic and random properties of chaotic mapping, the generated random numbers are deterministic when the initial values of the chaotic mapping are known in advance. The local end and the control center use chaotic mapping to construct the same random numbers, and the measurement matrix ***Φ*** is constructed by these random numbers according to the same rules.

**Figure 4:**
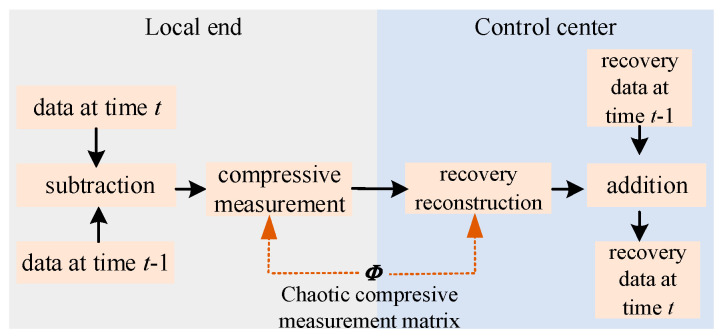
Compressed data acquisition principle based on compressive measurement of data difference.

Therefore, this paper is based on the chaotic measurement matrix to measure data difference compressively, and forms the compressed data acquisition method of the power system. The principle of compressed data acquisition at any time *t* is shown in Figure 4.

This paper proposes a compressed data acquisition method based on chaotic compressive measurement matrix, which needs to solve two key problems. The first problem is the construction and optimization of the chaotic compressive measurement matrix. The optimized chaotic compressive measurement matrix is a deterministic matrix related to specified parameters, and its compression performance is consistent with that of classical random measurement matrices. The second problem is the implementation of data acquisition based on compressed measurement results of data difference. By using the optimized chaotic compressive measurement matrix, the local data difference of the power system is compressed, and the control center can accurately calculate the original monitoring data of the power system according to the compressed data.

## 3. Compressed Data Acquisition Based on Chaotic Measurement Matrix

This section starts from the chaos mapping and studies a construction method for measurement matrix that transforms chaos mapping into chaotic compressed measurement matrix elements. Subsequently, the optimization is applied to enhance the compression performance of the chaotic measurement matrix. Finally, a compressed data acquisition method for power system is established based on the optimized chaotic compressed measurement matrix.

### 3.1. Chaotic Compressive Measurement Matrix

#### 3.1.1. The Basic Structure of Chaotic Compressive Measurement Matrix

In the selection of chaotic maps for constructing the compressed measurement matrix ***Φ***, this paper adopts a two-dimensional discrete chaotic map called Cat mapping, proposed by Arnold [26]. The specific expression of the Cat map is as follows:

(2) $$\left[\begin{array}{c} x_{n+1}\\ y_{n+1} \end{array}\right]=\left[\begin{array}{cc} 1 & a\\ b & ab+1 \end{array}\right]\left[\begin{array}{c} x_{n}\\ y_{n} \end{array}\right]\left(\mathrm{mod}1\right)$$

where *x_n_* and *y_n_* represent the values of sequences ***x*** and ***y*** after *n* transformations, *a* and *b* are mapping parameters, and mod 1 denotes the integer part of the real number.

The basic characteristic of the measurement matrix ***Φ*** is that its internal elements are composed of independent random numbers. The basic characteristic of the Cat mapping is that the generated sequence has randomness. Therefore, idea of constructing the compressive measurement matrix ***Φ*** based on the Cat mapping in this paper is to sample the sequence ***x*** of the Cat mapping and make the sampled data independent, and then construct the chaotic compressive measurement matrix ***Φ*** based on these independent sampled data. The expression of ***Φ*** is shown in (3) and (4):

(3) $$\Phi (i,j)=(c_{(i-1)\times N+j}),\;\;1\leq i\leq M,1\leq j\leq N$$

(4) $$c_{i}=x_{k_{0}+(i-1)\times l}\;\;i=1,2,3...$$

where *c_i_* is the sampled value of sequence ***x***, *k*_0_ is the sampling starting point, and *l* is the sampling interval.

Therefore, constructing the chaotic measurement matrix ***Φ*** requires calculating the sampling parameters *k*_0_ and *l* in order to ensure that the sampled elements have mutual independence.

#### 3.1.2. Parameter Calculation of Chaotic Compressive Measurement Matrix

This paper determines the parameters *l* and *k*_0_ based on the independence test method described in [27]. The basic idea of this method is to transform a random sequence with an unknown distribution into a standard normal distribution random sequence, and then perform an independence test. Therefore, in order to determine *l* and *k*_0_ that satisfy the independence requirement, the sequence *x_n_* is iteratively transformed into a standard normal distribution random sequence for a total of iterations *k*_0_ and iterations *k*_0_ + *l*, respectively, before conducting the independence test. The specific process is as follows:

Step 1: Set the initial parameters.

The initial parameters of Cat mapping are *a* = 1, *b* = 1, *y*_0_ = 1.

Step 2: Generate independent and identically distributed random sequences ***P*** and ***Q***.

Firstly, an *n*-dimensional random sequence ***X***_0_ = [*x*_0.1_, *x*_0.2_,…, *x*_0.*n*_] satisfying (0,1) uniform distribution is generated, and *x*_0.*i*_ (*i* = 1, 2,…, *n*) is taken as *x*_0_ of the Cat mapping. Next, assign values to *k_0_* and *l*. Finally, calculate the results of *k*_0_ iterations as sequence ***P***, and the results of *k*_0_ + *l* iterations as sequence ***Q***. The calculation expressions for each element in ***P*** and ***Q*** are as follows:

(5) $$\left\{\begin{array}{l} p_{i}=x_{k_{0}}{|}_{x_{0}=x_{0,i}},i=1,2,...,n\\ q_{i}=x_{k_{0}+l}{|}_{x_{0}=x_{0,i}},i=1,2,...,n \end{array}\right.$$

(6) $$n\geq \frac{In(2/\alpha )}{2{\left(\Delta \alpha \right)}^{2}}$$

where α is the significance level and Δα is the maximum allowable absolute error between the experimental cumulative distribution function and the theoretical distribution function.

Step 3: Transform ***P*** and ***Q***.

Obtain two standard normal distribution random sequences ***U*** and ***V*** from sequences ***P*** and ***Q***, using the following calculation expression:

(7) $$\left\{\begin{array}{l} \left[p\right]\to \left[p^{\prime }=F_{eP}\left(p\right)\right]\to \left[u=g\left(p^{\prime }\right)\right]\\ \left[q\right]\to \left[q^{\prime }=F_{eQ}\left(q\right)\right]\to \left[v=g\left(q^{\prime }\right)\right] \end{array}\right.$$

where *F_eP_*(*p*) and *F_eQ_*(*q*) are the experimental cumulative distribution functions of ***P*** and ***Q*** respectively, and g(.) is the inverse function of the standard normal distribution function.

Step 4: Establish the hypothesis.

Hypothesis *H*_0_ is ***P*** and ***Q*** are independent and hypothesis *H*_1_ is ***P*** and ***Q*** are not independent.

Step 5: Calculate the test value.

The calculation expression of the test value *T* is as follows:

(8) T=r(n–2)(1–r2)

(9) $$r=\sum_{i=1}^{n}\left(u_{i}–\;\bar{u})(v_{i}–\;\bar{v}\right)/\sqrt{\left[\sum_{i=1}^{n}{\left(u_{i}–\;\bar{u}\right)}^{2}][\sum_{i=1}^{n}{\left(v_{i}–\;\bar{v}\right)}^{2}\right]}$$

where *r* is the correlation coefficient between ***U*** and ***V***. If the hypothesis *H*_0_ is true, the test value *T*~*T*(*n* − 2).

Step 6: Statistical inference.

The rejection region of hypothesis *H*_0_ is |*T*| > *T_α_*_/2_, which represents the interval of statistic that ***P*** and ***Q*** are not independent. If |*T*| ≤ *T_α_*_/2_ is not in the rejection region and ***U*** and ***V*** follow a joint normal distribution, hypothesis *H*_0_ is accepted.

Repeat steps 1–6 to find the *l* and *k*_0_ that satisfy the independence requirement. Next, *l* and *k*_0_ are substituted into (3) and (4) to obtain the chaotic measurement matrix ***Φ***.

### 3.2. Optimization of Chaotic Compressive Measurement Matrix

The compression performance of the chaotic compressive measurement matrix ***Φ*** constructed in Section 3.1 is still not comparable to the classical and well-recognized random measurement matrix, and it also cannot meet the application requirements of power systems. Therefore, further optimization is needed for the chaotic compressive measurement matrix ***Φ***.

#### 3.2.1. Column Coherence of Compressive Measurement Matrix

According to the requirements of compressed sensing principle, the sensing matrix ***Θ*** = ***ΦΨ*** must satisfy the RIP. Since ***Ψ*** is processed as a unit matrix in this paper, it is only necessary for the chaotic measurement matrix ***Φ*** to satisfy the RIP. However, the calculation process for verifying the RIP is very complex. Therefore, it can be replaced by analyzing the column coherence of ***Φ*** [28,29].

The calculation expression of the column coherence coefficient *μ* for any compressive measurement matrix ***Φ*** is as follows:

(10) $$\mu =\underset{i\neq j}{max}\left|\langle {\hat{\Phi }}_{i},{\hat{\Phi }}_{j}\rangle \right|=\underset{i\neq j}{max}\left|g_{ij}\right|$$

where $\hat{\Phi }$ is the unitization matrix of ***Φ***, and *g_ij_* is an off-diagonal element of gram matrix $G={\hat{\Phi }}^{T}\hat{\Phi }$.

For $\hat{\Phi }$, *μ* needs to satisfy the following constraints:

(11) $$\left\{\begin{array}{l} \mu_{L}\leq \mu \leq 1\\ \mu_{L}=\sqrt{\left(N–M)/[(N–1)M\right]} \end{array}\right.$$

The smaller the value of *μ*, the stronger the compression performance of $\hat{\Phi }$. Therefore, *μ* can be used as an important indicator for judging the performance of $\hat{\Phi }$.

#### 3.2.2. Optimization of Compressive Measurement Matrix

The overall coherence coefficient of $\hat{\Phi }$ is represented by the sum of squares of the non-diagonal elements *g_ij_* of matrix ***G***, denoted as *μ_w_* in this paper. Based on the fundamental characteristics of matrix ***G*** [30,31], the calculation expression of *μ_w_* is as follows:

(12) $$\mu_{w}=\sum_{i\neq j}{\left(g_{ij}\right)}^{2}=\sum_{k=1}^{M}{\left(I_{k}\right)}^{2}-\sum_{k=1}^{N}{\left(g_{ii}\right)}^{2}$$

where *g_ii_* is the diagonal element of gram matrix ***G***, and *I_k_* (*k* = 1 − *M*) is the eigenvalues of gram matrix ***G***.

Optimizing *μ_w_* not only reduces the column correlation coefficient of the matrix, but also improves the overall performance of compression. Since the diagonal elements *g_ii_* of matrix ***G*** have a value of 1 and the sum of *I_k_* (*k* = 1:*M*) is *N*, an optimization equation can be established with the goal of minimizing *μ_w_*, as shown in (13).

(13) $$\left\{\begin{array}{l} min\mu_{w}=min\left(\sum_{k=1}^{M}{\left(I_{k}\right)}^{2}-N\right)\\ s.t.\sum_{k=1}^{M}I_{k}=N \end{array}\right.$$

From (13), it can be seen that the process of solving the optimization equation is the same as the process of finding the optimal *I_k_*. The steps for finding the optimal *I_k_* are as follows:

Step 1: Normalize the columns of matrix ***Φ*** to obtain matrix $\hat{\Phi }$ and the Gram matrix $G={\hat{\Phi }}^{T}\hat{\Phi }$.

Step 2: Perform eigenvalue decomposition on ***G*** to obtain ***G*** = ***VHV***^T^. Next, set the non-zero elements on the diagonal of matrix ***H*** to *N/M* to obtain matrix ***H***’.

Step 3: Decompose the diagonal matrix ***H***’ to obtain ***H***’ = ***L***^T^***L***.

Step 4: Substitute ***H***’ = ***L***^T^***L*** into ***G*** to obtain $\hat{G}=VL^{T}LV^{T}$.

Step 5: Let $\Phi^{\prime }=LV^{T}$, and calculate the Gram matrix $G_{new}={\Phi^{\prime }}^{T}\Phi^{\prime }$ of $\hat{G}$.

Step 6: Calculate the sum of squares of the off-diagonal elements of $G_{new}$, denoted as *μ_w_*. If it satisfies Equation (14), then terminate the calculation, and *I_k_* is optimal. At this point, the optimized chaotic compressive measurement matrix is $\Phi^{\prime }=LV^{T}$. Otherwise, return to Step 2.

(14) $$|\mu_{w}-\sum_{k=1}^{M}{\left(N/M\right)}^{2}-N|<0.1$$

### 3.3. Compressed Data Acquisition Process Based on Chaotic Measurement Matrix

This paper proposes a power system compressed data acquisition method to measure differences in monitoring data based on chaotic compressive measurement matrix. Through the calculation of the data difference between adjacent time and the chaotic compressive measurement at the local end, as well as the difference recovery reconstruction and data acquisition calculation at the control center, the compressed acquisition of the monitoring data is realized.

If the number of local monitoring nodes is *n*, the monitoring data at any time *t* is denoted as ***s****^t^*:

(15) $$s^{t}=[s_{1}^{t},s_{2}^{t},...,s_{n}^{t}{]}^{T}$$

The basic requirement for compressed acquisition of data ***s****^t^* based on chaotic measurement matrix is that the control center can accurately obtain all local data only by transmitting compressed data from the local end. The compressed acquisition process of power system data used in this paper is as follows:

Step 1: Calculate data difference.

Data difference calculation is undertaken to obtain sparse and compressible data. The calculation expression for the data difference vector Δ***s****^t^* between adjacent times is as follows:

(16) $$\Delta s^{t}=s^{t}-s^{t-1}$$

To ensure that the difference vector Δ***s****^t^* is sufficiently sparse, identify the elements in Δ***s****^t^* with absolute values smaller than the set threshold. After calculating the average value $\Delta {\bar{s}}_{t}$ of these elements, set them to zero.

Step 2: Compressive measurement of data difference.

The optimized chaotic measurement matrix ***Φ***’ is used to compress the processed difference Δ***s****^t^.* The compressed data ***y****^t^* with length of *m* are generated from the Δ***s****^t^* with length *n* according to the following calculation expression:

(17) $$y^{t}=\Phi^{\prime }\Delta s^{t}$$

The compressed ***y**^t^* and the $\Delta {\bar{s}}_{t}$ obtained in step 1 are transmitted to the control center from local end. The amount of data transmitted is *m* + 1. Compared to traditional data acquisition methods that require the complete transmission of *n* data, the method in this paper significantly reduces the amount of data transmission due to *m* being much smaller than *n*.

Step 3: Recovery reconstruction of data difference.

In the control center, the recovery of ***y**^t^* from the local end is performed to obtain the reconstructed value $\Delta s_{r}^{t}$. The reconstruction model can be represented by an optimization equation, expressed as

(18) $$\Delta s_{r}^{t}=argmin\;{\left\|\Delta s_{r}^{t}\right\|}_{1}\;\;s.t.\;y^{t}=\Phi^{\prime }\Delta s^{t}$$

where $\Delta s_{r}^{t}$ represents the data difference between adjacent times obtained by the control center, and $\;{\left\|.\right\|}_{1}$ represents the *l*_1_ norm.

Equation (18) can be calculated by the classical orthogonal matching pursuit algorithm.

Step 4: Calculate the acquisition data.

In order to obtain the original data values from the compressed data, the control center calculates the acquisition data $s_{r}^{t}$ at any time *t* according to the obtained $\Delta s_{r}^{t}$ and $\Delta {\bar{s}}_{t}$. The data acquisition calculation includes the following two aspects:

Firstly, calculate the original data based on the reconstructed difference vector $\Delta s_{r}^{t}$. This involves adding the $\Delta s_{r}^{t}$ at time *t* with the previous data acquisition values at time *t* − 1 to obtain the complete acquisition data at time *t*.

Secondly, reduce the error generated during the acquisition process. On one hand, in order to minimize the error caused by approximating the elements of the difference vector Δ***s**^t^* to 0, the 0 elements generated by the approximation in $\Delta s_{r}^{t}$ need to be corrected using $\Delta {\bar{s}}_{t}$. On the other hand, in order to reduce the cumulative error generated during the acquisition process, it is necessary to transmit a complete set of local data at a certain acquisition period, that is, to define the acquisition interval *T_e_* for transmitting a complete set of data once.

Therefore, the calculating expressions of the acquisition data at any time *t* are

(19) $$s_{ri}^{t}=\left\{\begin{array}{l} s_{ri}^{t-1}+\Delta s_{ri}^{t},\begin{array}{c} \;\;\Delta s_{ri}^{t}\neq 0\&t\neq t_{0}+kT_{e} \end{array}\\ s_{ri}^{t-1}+\Delta {\bar{s}}_{t},\begin{array}{c} \;\;\Delta s_{ri}^{t}=0\&t\neq t_{0}+kT_{e} \end{array}\\ s_{i}^{t},\begin{array}{c} \;\;t=t_{0}+kT_{e} \end{array} \end{array}\right.,k=1,2,\ldots$$

where *t*_0_ is the initial acquisition time, and *T_e_* is the regular time interval for transmitting the complete local data.

The calculation result from (19) represents the data acquisition value at the control center. Based on the above steps, 1–4, the compressed acquisition of power system data from the local end to the control center is realized.

## 4. Case Studies

**Figure 5:**
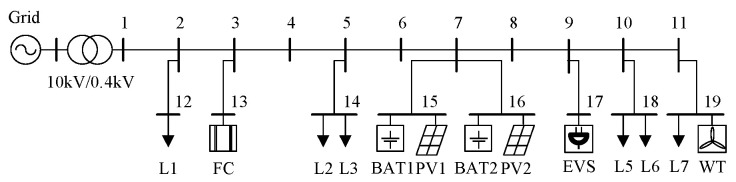
Microgrid structure diagram.

To verify the correctness of the compressed data acquisition method for power systems based on chaotic compressive measurement studied in this paper, a 19-bus microgrid system is taken as a case. The original parameters of the lines and loads can be found in reference [32]. In order to incorporate the operational characteristics of a new power system into the case, electric vehicle charging stations (EVSs), fuel cells (FCs), wind turbines (WTs), and two sets of photovoltaic energy storage systems (PV1 + BAT1, PV2 + BAT2) are integrated based on the original parameters. The specific circuit structure and the connection positions of each device are shown in Figure 5.

### 4.1. Case Data Samples

The correctness of the proposed method is verified using the acquired data of three-phase voltage values from 19 buses. The amount of data acquired at a single time is 57. To obtain multiple sets of data acquired over continuous time, a power flow calculation tool is used to calculate the three-phase voltage values every 5 min for a period of 24 h. Based on the calculation results, a data sample with a size of 57 × 288 is constructed.

The original case used as a standard example lacks 24 h calculation parameters. Before conducting power flow calculations, the operating parameters of each load and device change every 5 min are constructed. The 24 h load data are based on the original load, and their change trend refers to the load data of the WESTERN area given by the PJM company in the United States. The 24 h photovoltaics data come from the photovoltaic output on a certain day in the WESTERN region. The 24 h data for electric vehicle charging stations and wind turbines are generated based on their mathematical models [33,34]. The power flow calculation tool is used to calculate the three-phase voltage value at a time interval of 5 min within 24 h, and the voltage data sample for this example is obtained.

### 4.2. The Obtained Chaotic Compressive Measurement Matrix

**Figure 6:**
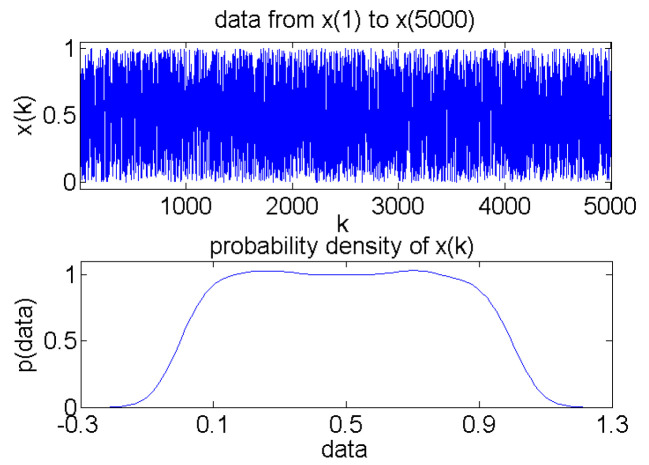
Values and probability density curves of *x_n_.*

Under the conditions of *a* = 1, *b* = 1, and initial values *x*_0_ = 0.09, *y*_0_ = 1 of Cat mapping, the 1–5000 data [*x*_1_, *x*_5000_] and their probability density for the sequence *x_n_* are shown in Figure 6.

It can be seen from Figure 6 that chaotic mapping, when the initial value is determined, can obtain deterministic data with random distribution characteristics. Therefore, the chaotic measurement matrix can also be constructed with deterministic characteristics according to certain rules, under the condition that the mapping initial parameters are determined.

The construction method for the chaotic compressive measurement matrix based on Section 3.1 is used to obtain the sampling parameters k0 and l of the Cat mapping *x_n_* sequence, which are *k*_0_ = 500 and *l* = 23, respectively. Utilizing the sampled data for the *x_n_* sequence, the chaotic measurement matrix ***Φ*** is constructed based on (3) and (4), according to the values of length n and compression measurement number m of the data to be acquired.

The optimization method based on Section 3.2 for the chaotic compressive measurement matrix further optimizes the ***Φ***, and uses the optimized chaotic measurement matrix as the measurement matrix for the compressed data acquisition method.

To demonstrate the excellent compression performance of the optimized chaotic measurement matrix ***Φ***′, we calculate its column coherence coefficient *μ* and compare it with the *μ* of the original ***Φ*** and the *μ* of the classical Gaussian random measurement matrix ***Φ****_N_*. The values of *μ* for these three measurement matrices, all of size 30 × 10, are shown in Table 1.

From Table 1, it can be seen that the optimized matrix ***Φ***′ has a smaller *μ*, with a value of approximately 56% of the un-optimized ***Φ***. The compression performance has been greatly improved. In addition, the *μ* of the optimized matrix ***Φ***′ is slightly smaller than that of the classical and high-performance Gaussian measurement matrix ***Φ****_N_*, indicating that the compression performance of the optimized matrix can be comparable to that of the classical random measurement matrix, which fully meets the application requirements.

### 4.3. Compression Performance Analysis of Compressed Data Acquisition Method

In order to illustrate the compression performance of the compressed acquisition method studied in this paper, the compression ratio (CR) is used as the analysis index. The calculation formula for CR is

(20) $$\mathrm{CR}=\frac{N_{\mathrm{CS}}}{N_{\mathrm{OS}}}\times 100\%$$

where *N*_OS_ represents the amount of local data, and *N*_CS_ represents the amount of transmitted data. The amount of data transmitted by the method proposed in this paper is *m* + 1.

#### 4.3.1. Compressive Measurement Results of Local Data

**Figure 7:**
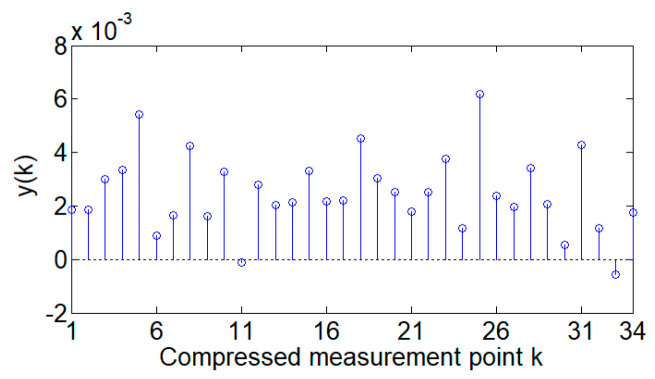
The measured compressed data ***y***.

Based on the method proposed in this paper, ***Φ***′ is used to compress the voltage difference at any adjacent time to obtain the compressed measurement data ***y***. In this case, the CR is set to 70%, which means that the voltage difference data with a size of 57 are compressed to obtain compressed data ***y*** with a size of 39. Taking an example of the compressed data ***y*** obtained by measuring matrix ***Φ***′ for a certain voltage difference, the compression measurement data ***y*** of the compressed acquisition method proposed in this paper are shown in Figure 7.

**Figure 8:**
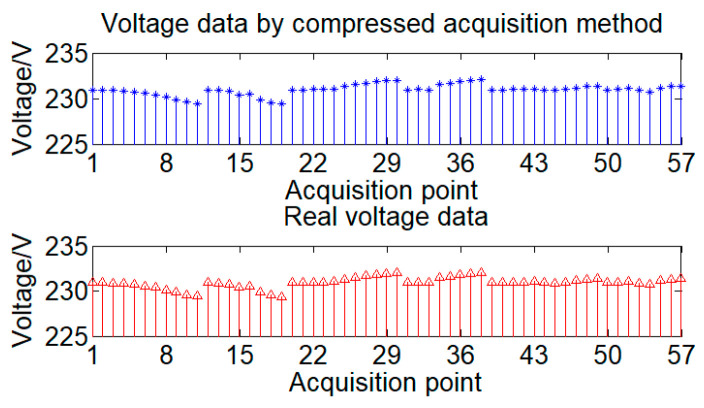
Comparison of compressed acquisition data and real data.

To demonstrate that the original acquisition data can be obtained from the compressed data ***y*** using the method described in this paper, a comparison between the compressed acquisition results and the actual data is shown in Figure 8.

From Figure 7 and Figure 8, it can be seen that the compression measurement number of 39 is less than the data length of 57. Compressing the data difference can obtain a smaller quantity of compressed data, greatly reducing the pressure of data transmission. Moreover, the acquisition results obtained from the compressed data ***y*** are basically the same as the actual data values, indicating that compressed measurement can preserve a large amount of information from the original data, achieving high-quality compression and demonstrating the excellent compression performance of the method proposed in this paper.

#### 4.3.2. Compression Performance Analysis of Compressed Acquisition Method

In order to illustrate the compression performance of the compressed acquisition method in this paper further, we calculated the minimum compressive measurement number *m* required to compress the voltage difference, which is the minimum *m* to ensure the correct recovery of data. We also calculated the values of the amount of transmitted data and CR based on *m*. The calculation results are shown in Table 2.

From the calculation results in Table 2, it can be seen that the minimum number of the compressive measurement *m* required to ensure correct data recovery is 33. The CR reaches 57.9%, achieving a significant compression of the data and greatly reducing the data acquisition pressure on the power system. Compared to traditional data acquisition methods that involve complete data transmission and achieve a data transfer number up to 57, the method proposed in this paper has made significant progress, with a data transfer number of 34, which is less than 60% of the traditional methods. The calculation results demonstrate that the method proposed in this paper can significantly reduce data transmission numbers and possesses excellent compression performance, making it highly suitable for the rapid acquisition of large amounts of monitoring data in new power systems.

### 4.4. Accuracy Analysis of Compressed Data Acquisition Method

In order to illustrate the acquisition accuracy of the compressed acquisition method proposed in this paper, the maximum relative error (MRE) between each element of the compressed acquisition data and the original data is taken as the evaluation index. The calculation formula is as follows:

(21) $$\mathrm{MRE}=\max[abs(s_{r}–s_{0})/s_{0}]\times 100\%$$

where ***s***_0_ is the original data, and ***s****_r_* is the compressed acquisition data.

#### 4.4.1. Acquisition Accuracy of Different Compressed Acquisition Methods

**Figure 9:**
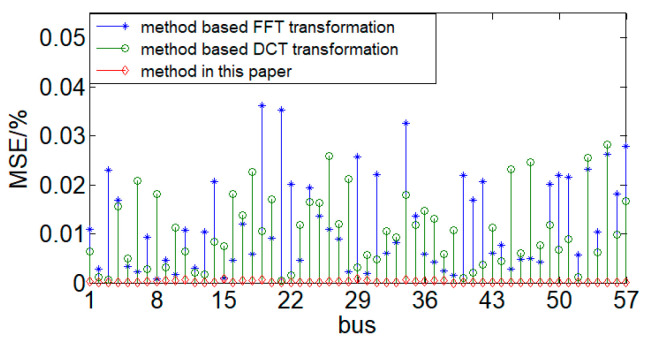
The accuracy of different compressed acquisition methods.

By comparing our approach with spatial-transformation-based compressed acquisition methods, we analyze the accuracy of different compressed sensing methods. Using CR = 57.9% from Table 2, we perform compressed data acquisition on the same voltage data using two different spatial transformations (FFT transformation and DCT transformation). The acquisition results are compared with those obtained using the method presented in this paper under the condition of CR = 57.9%. The MRE for the three methods is calculated, as shown in Figure 9.

As shown in Figure 9, with the same voltage data and CR, the data acquisition accuracy of the compressed acquisition method proposed in this paper is the highest, with MRE ≈ 0. In contrast, the accuracy of the other two data acquisition methods is significantly lower than that of the proposed method. This is because the method proposed in this paper directly measures the information in the data based on the measurement matrix, whereas spatial transformation methods, which retain the main information, do not achieve the same level of accuracy. The proposed method can greatly improve the acquisition accuracy.

#### 4.4.2. Acquisition Accuracy of Different *m*

**Figure 10:**
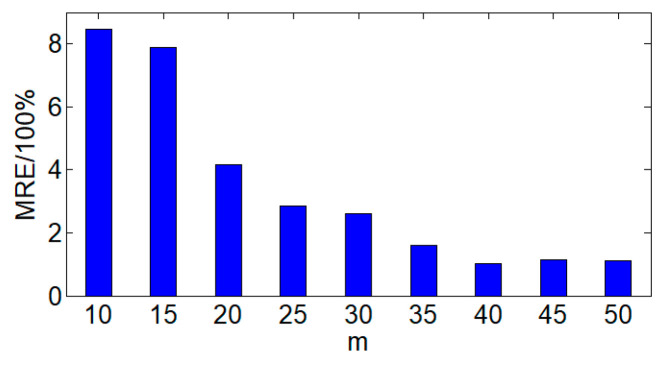
MRE with different *m*.

By changing the compressive measurement number *m* of the data and setting a regular time interval *T_e_* for transmitting the complete acquisition data to 288, which means that the complete data are transmitted every 24 h, the impact of the cumulative error on the data acquisition accuracy is analyzed. Under different *m* conditions, starting from the transmission of the complete data once, the MRE calculation results for the 288th data acquisition is shown in Figure 10.

It can be seen from Figure 10 that as the *m* increases, the MRE of the acquisition data gradually decreases. When *m* < 30, the MRE is relatively large (> 3%), indicating that smaller *m* cannot achieve accurate data acquisition. The value of *m* in the acquisition method should not be too low. Furthermore, in Section 4.3.2 the minimum *m* is calculated as 33, which ensures the correct recovery of data within a certain accuracy. When *m* > 40, the variation of MRE stabilizes at around 1%. At this time, the data acquisition accuracy becomes stable and at a relatively high level of precision. In addition, the stable trend of the MRE changes indicates that it is unnecessary to sacrifice the compressive measurement number to ensure data accuracy, which is more beneficial for the practical application of new power systems.

#### 4.4.3. Acquisition Accuracy of Different *T_e_*

**Figure 11:**
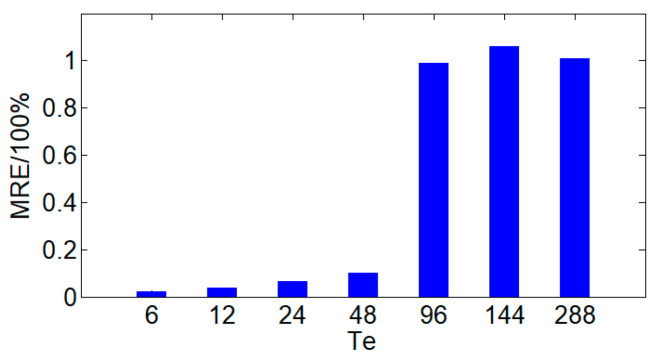
MRE with different *T_e_*.

By changing the regular time interval *T_e_* for transmitting the complete acquisition data, the impact of cumulative error on data acquisition accuracy is analyzed. Under different *T_e_* conditions, the MRE calculation results of the last acquisition data in each *T_e_* period are shown in Figure 11.

From Figure 11, it can be seen that as *T_e_* increases, the MRE of the last data acquisition within each *T_e_* period gradually increases. Furthermore, even when *T_e_* is increased to 288, which means transmitting complete data every 24 h, the MRE is still around 1%, indicating that the proposed compressed acquisition method in this paper has high accuracy. When *T_e_* < 48, that is, when the interval for transmitting complete data is no more than 4 h, the MRE is close to 0, indicating that the data acquisition accuracy is already very high. This suggests that reducing *T_e_* can significantly reduce relative errors and improve data acquisition accuracy. In addition, when *T_e_* < 48, the MRE tends to stabilize, indicating that the proposed method in this paper does not need to reduce *T_e_* to improve data acquisition accuracy, thereby, significantly, avoiding an additional burden on the communication system caused by transmitting complete data.

In the application process of the new power system, it is possible to set a reasonable cycle *T_e_* according to the actual application requirements, ensuring the high accuracy of data acquisition without adding pressure to data transmission.

## 5. Conclusions

This paper combines the advanced concept of compressing data without compressing information based on the compressive sensing principle with the data acquisition of the power system. It innovatively proposes a compressed data acquisition method based on the chaotic measurement matrix, which not only ensures the accuracy of data acquisition, but also greatly reduces the amount of data transmission. The main conclusions of this paper are as follows:

1. The key issue in combining the compressed sensing principle with power data acquisition is that the same compressive measurement matrix is required for the compressive measurement at the local end and the recovery reconstruction at the control center. Therefore, the compressive measurement matrix needs to have deterministic characteristics to avoid the communication of the measurement matrix between the two ends.
2. The random, yet deterministic nature of chaotic mapping is highly compatible with the requirements of the compressive measurement matrix. Based on the construction method of the deterministic chaotic compressive measurement matrix studied in this paper, when the initial value of Cat mapping is known, both the local end and the control center can obtain the same measurement matrix using this method to complete the compressive measurement and recovery reconstruction of data.
3. The difference between adjacent monitoring data in the power system itself has compressibility. It is feasible to construct a compressed data acquisition method for the power system using the compressive measurement of the data difference. In order to reduce the cumulative error of the compressed acquisition method, it is necessary to further improve the sparsity of the data differences and set a certain period for transmitting complete local data.
4. The compression ratio and accuracy of the power system compressed data acquisition method proposed in this paper are very stable, and it is not necessary to increase the number of compressive measurements or reduce the complete data transmission cycle to ensure data accuracy. However, there is a lower limit to the number of compressive measurements in this method, and it is advisable to avoid using a small number of compressive measurements in practical applications.

## Figures and Tables

**Table 1:** The *μ* of three measurement matrices.

Measurement Matrice	*Φ*′	*Φ*′	*Φ* *_N_*
*μ*	0.5180	0.9110	0.6322

**Table 2:** Key items calculation results to ensure correct data recovery.

Item	Minimum *m*	Transmitted Amounts	CR
Numerical results	33	34	57.9%

## Data Availability

Dataset available on request from the authors.
